# Luteolin improves precancerous conditions of the gastric mucosa by binding STAT3 and inhibiting LCN2 expression

**DOI:** 10.7150/ijbs.111636

**Published:** 2025-05-07

**Authors:** Xinyu Hao, Shouli Yuan, Jing Ning, Yuanfei Zhou, Yanfei Lang, Xiurui Han, Qiao Meng, Ying Xiong, Rongli Cui, Yueqing Gong, Chao Ma, Weichao Xu, Yangang Wang, Xiaohuan Guo, Chu Wang, Jing Zhang, Weiwei Fu, Shigang Ding

**Affiliations:** 1 Department of Gastroenterology, Peking University Third Hospital, Beijing, China; 2 Beijing Key Laboratory for Helicobacter Pylori Infection and Upper Gastrointestinal Diseases (BZ0317), Beijing, China; 3 Peking-Tsinghua Center for Life Sciences, Academy for Advanced Interdisciplinary Studies, Peking University, Beijing, China; 4 Synthetic and Functional Biomolecules Center, Beijing National Laboratory for Molecular Sciences, Key Laboratory of Bioorganic Chemistry and Molecular Engineering of Ministry of Education, College of Chemistry and Molecular Engineering, Peking University, Beijing, China; 5 Department of Gastroenterology, Hebei Hospital of Traditional Chinese Medicine, Shijiazhuang, China; 6 Department of Gastroenterology, Beijing University of Chinese Medicine Third Affiliated Hospital, Beijing, China; 7 Institute for Immunology, Tsinghua University, Beijing, China; 8 Peking University Chengdu Academy for Advanced Interdisciplinary Biotechnologies, Chengdu, China

**Keywords:** Gastric cancer, Precancerous gastric conditions, Spasmolytic polypeptide-expressing metaplasia, Luteolin, LCN2, STAT3

## Abstract

Inhibition of malignant transformation from the precancerous stage has important clinical value for the prevention of gastric cancer. Here, we report a strategy to inhibit precancerous gastric conditions by Luteolin (Lut). Lut treatment resulted in remarkable resistance to oxyntic atrophy, spasmolytic polypeptide-expressing metaplasia (SPEM), and gastric mucosal injury in tamoxifen (TAM)-treated mice, chenodeoxycholic acid-treated rats, and human organoids. Mechanism study suggested that LCN2 expression was upregulated in the SPEM mucosa and downregulated after Lut treatment. LCN2 blocking suppressed TAM-induced oxyntic atrophy and metaplasia and partially counteracted the effect of Lut. Quantitative chemoproteomics identified that Lut bound to STAT3 and inhibited its phosphorylation. Functional experiments using STAT3 inhibitors and epithelial cell-specific *Stat3* deficient mice showed that STAT3 inhibition and deletion attenuated the beneficial effects of Lut. Our data supported that Lut might be a therapeutic candidate for the treatment of gastric mucosal injury by binding to STAT3 and thereby inhibiting the STAT3/LCN2 axis.

## Introduction

Gastric cancer (GC) is diagnosed in approximately one million new patients annually^1^ and usually occurs in the precancerous stage including atrophic gastritis (AG), spasmolytic polypeptide-expressing metaplasia (SPEM), intestinal metaplasia (IM), and intraepithelial neoplasia^2^. SPEM and IM are two types of parallel metaplasia that are considered crucial for the development of GC. In particular, SPEM, which is characterized by the presence of pyloric adenoid glands in the gastric corpus, is considered a key pretumor event for a possible malignant transformation, and a potential cell source for IM, intraepithelial neoplasia, and eventual adenocarcinoma^3^. SPEM is located in areas adjacent to the cancer and was subsequently identified in biopsy or excision samples of gastric stump cancer^4^. Currently, there is no effective therapeutic strategy that targets these precancerous stages. Therefore, screening for new effective treatment strategies at the precancerous gastric conditions has important scientific significance and clinical value in the prevention of GC.

Luteolin (3′,4′,5,7-tetrahydroxy flavones, Lut), a natural flavonoid widely found in many medicinal herbs, has shown marked anti-inflammatory, antioxidant and anti-apoptotic properties^5^. In the digestive system, Lut can inhibit the proliferation of GC and pancreatic cancer cells and has the potential to prevent liver fibrosis and cancer, as well as to relieve ulcerative colitis^6^-^8^. We previously reported that Lut has antitumor activity, as confirmed by organoids derived from patients with GC. Compared with clinical drugs, Lut exhibits a stronger anti-GC effect than carboplatin and with similar effects as norcantharidin^9^. However, whether Lut can eliminate precancerous gastric conditions requires further confirmation.

STAT3 is a classic transcription factor that promotes the transcription of genes related to proliferation, apoptosis, survival, and metastasis. STAT3 is over-activated in many human malignancies, including gastrointestinal tumors, thereby accelerating tumor progression, metastasis, and drug resistance^10^. C57BL/6 mice infected with *Helicobacter pylori* (*H. pylori*) develop SPEM six months after infection, accompanied by increased expression of IL-6 and p-STAT3^11^. Moreover, STAT3 has also shown an increase in concentration during the transition from chronic gastritis to IM^12^. Lipocalin 2 (LCN2) is a small secreted glycoprotein with a molecular weight of 24-25 kDa that binds to ferriferite. Secreted by a variety of cells, LCN2 is an important biomarker of inflammation, infection, and organ damage and is considered a promoter of cancer progression^13^. Clinical samples and animal models have confirmed that LCN2 expression increased significantly in the gastric mucosa of individuals and mice with *H. pylori*^14^. STAT3 could bind to the promoter region of the* Lcn2* gene and regulate its expression^15^. However, the effects of STAT3 and LCN2 in the occurrence and development of precancerous conditions in the gastric mucosa remain unclear.

In the present study, we found that Lut treatment resulted in remarkable resistance to oxyntic atrophy, SPEM, and gastric mucosal injury in both animal models and human organoids. Further mechanism study revealed that LCN2 might be a new therapeutic target for precancerous gastric conditions, and demonstrated that STAT3 was a direct target of Lut. We identified Lut as a new therapeutic candidate for gastric mucosal injury that could inhibit the STAT3/LCN2 axis by binding to STAT3.

## Materials and Methods

### Qualitative Analysis by HPLC/QTOF-HR-MS/MS

Liquid chromatography-mass spectrometry analysis was used to identify the chemical composition of Yinglian Hewei and Shiwei Baihe granules^16^,^17^. About 200 mg of Yinglian Hewei granules (Hebei Hospital of Traditional Chinese Medicine, Cat#20210307) and Shiwei Baihe granules (Hebei Hospital of Traditional Chinese Medicine, Cat#20210401) were weighed, 1 mL of 80% methanol solution and grinding beads were added and ground for 5 min. The mixture was swirled for 10 min, centrifuged at 4 ℃ for 10 min with centrifugal force of 20000 ×*g*, and then the supernatant was filtered and analyzed. The data collected by high-resolution liquid and mass spectrometry were preliminarily sorted using CD2.1 (Thermo Fisher) and then searched and compared in databases (mzCloud, mzVault). The detection conditions are listed in Table S1.

### Animal and treatment protocol

Overall, 6-8-week-old male C57BL/6 and 6-8-week-old male Sprague-Dawley rats were obtained from the Peking University Health Science Center (Beijing, China). *Stat3^fl/fl^* mice were kindly gifted by Prof. Xiaohuan Guo's lab^18^. *Stat3^fl/fl^* mice were crossed with *VillinCre* mice to generate *Stat3^fl/fl^VillinCre* mice. All animals were housed under specific pathogen-free conditions. All procedures involving experimental animals were approved by the Peking University Biomedical Ethics Committee (LA2021589) and were performed in accordance with the National Guidelines for Animal Usage in Research.

In the high-dose tamoxifen (TAM) model, mice were treated with 20 mg/kg or 40 mg/kg Lut (MedChemExpress, HY-N0162) by gavage for 10 or 4 days and intraperitoneally injected with TAM (5 mg/20 g body weight; MedChemExpress, Cat#HY-13757A) dissolved in 10% ethanol and 90% sunflower oil for 3 days.

In the chenodeoxycholic acid (CDCA) model, rats were administered 20 mg/kg CDCA by gavage (Macklin, C804610-25 g) for 6 months and then treated with 40 mg/kg Lut for 3 months.

To detect Lut toxicity, mice were administered Lut by gavage for 10 consecutive days, and then serum and organs were collected, including liver, heart, spleen, kidney, and intestinal tract.

To deplete LCN2, mice were intraperitoneally injected with a 50 -μg dose of an anti-LCN2 (Novus Biologicals, Cat#AF1857) or an isotype IgG (Bio X Cell, Cat#BP0090) for 5 consecutive days.

To suppress p-STAT3, mice were administered 50 mg/kg C188-9(Selleck, Cat#S8605) by gavage for 7 consecutive days.

### Histological scoring of gastritis in the CDCA model

Six criteria are included in the mouse/rat gastric histological activity index (HAI): (1) inflammation, (2) epithelial defects, (3) oxyntic atrophy, (4) hyperplasia, (5) pseudopyloric metaplasia, and (6) dysplasia/neoplasia. Each of the six primary criteria was described using specific characteristics to assign a score on a scale 0-4^19^.

### Human organoids

Gastric corpus samples with metaplasia of the pyloric gland were obtained from patients undergoing endoscopy or treatment at the Third Peking University Hospital. Prior to sample acquisition, all patients provided written informed consent for the use of their clinical data and specimens, in accordance with the principles of the Declaration of Helsinki. The study was conducted in accordance with the national guidelines and was approved by the Ethics Committee of Peking University Third Hospital (Approval No.: IRB00006761-M2021434).

The samples were removed from the preservation solution and repeatedly rinsed with D-PBS (Solarbio, Cat#D1040) containing 100 U/mL penicillin-streptomycin-amphotericin B until the D-PBS was clear. The sample was then added to the enzymolysis reagent supplemented with Advanced DMEM/F12, Type I collagenase (Sigma-Aldrich, Cat#V900891-100MG), Type II collagenase (Gibco, Cat#17101015), and Type IV collagenase (Worthington, Cat#LS004188) and placed in a 37 °C water bath for 20 min. Subsequently, the solution was filtered through a 70-µm cell mesh sieve and centrifuged (300 ×*g*, 5 min). Gastric organoid medium (Kingculture TM Organoid Growth Medium, Cat#KCW-16-2) and Matrigel (R&D, Cat#3536-010-02) were mixed in a 1:1 ratio to resuspend the cells and subsequently inoculated into 24-well plates at 50 μL per well. After 30 min in a cell culture incubator at 37 °C with 5% CO_2_, 600 μL gastric organoid medium was added to each well, and the medium was changed every 3 days. For the Lut intervention, PDOs were digested into single cells and seeded in 24-well plates containing Matrigel. After 4 days, PDOs were treated with 20 μM Lut for 5 days at 37 ℃ in a 5% CO_2_ atmosphere, and 0.1% DMSO served as a negative control (NC).

### Cell lines

Human GES-1 and human GC cell lines were purchased from the Institute of Biochemistry and Cell Biology of the Chinese Academy of Sciences (Shanghai, China). Cells were cultured in an RPMI-1640 medium (Gibco, Cat#C11875500BT) supplemented with 10% fetal bovine serum (Gibco, Cat#10099141C), 100 units/mL of penicillin, and 100 mg/mL of streptomycin (Solarbio, Cat#P1400) at 37 °C in a humidified 5% CO_2_ atmosphere.

For the CDCA intervention, GES-1 cells were treated with 150 μM CDCA (Sigma-Aldrich, Cat#C9377-100MG) for 24 h after 2 h of serum starvation, and then with Lut treated for 24 h. To verify the inhibition efficiency of C188-9, AGS cells were given 10 μM C188-9 for 24 h.

### Human samples

Serum samples from CAG or IM patients before and after treatment with Chinese herbs were obtained from the Third Affiliated Hospital of Beijing University of Chinese Medicine. All samples were collected after obtaining informed consent from the patients and the experiment was approved by the Third Affiliated Hospital of Beijing University of Chinese Medicine (BZYSY-2024YJSKTPJ-39). Detailed information about the basic patient information and pathological types are listed in Table S2.

### Determination of the concentration of Lut in the blood

A 50 μL-blood sample was obtained, 150 μL ethanol was added, vortexed for 3 min, and centrifuged at 20,000 ×*g* at 4 ℃ for 20 min. The supernatant was spin dried at room temperature and dissolved with 50% acetonitrile/ddH_2_O. The Lut concentration was analyzed using an LC-SRM system that included an AB SCIEX 5500 triple-quadrupole mass spectrometer and a SHIMADZU DGU-20A liquid chromatography instrument with an Agilent column (Poroshell 120 EC-C18, 4.6 × 5 mm, 2.7 μm).

### Histology and immunostaining

Animal tissues were fixed with 4% paraformaldehyde (Santa Cruz Biotechnology, Cat#sc-281692) for 24 h and organoids were fixed with 4% paraformaldehyde for 12 h. A 3% agarose solution (HydraGene, Cat#R9012LE-100g) was prepared with TAE (Solarbio, Cat#T1061) and heated to boiling point in a microwave oven. After cooling slightly, the organoids were suspended and coagulated. Animal tissues and organoids were dehydrated with an alcohol gradient, embedded in paraffin, sectioned, and stained with hematoxylin and eosin (H&E). For immunohistochemical staining (IHC), paraffin sections were dried overnight at 37 °C, soaked in xylene, anhydrous ethanol, 95% ethanol, 80% ethanol and distilled water, then immersed in 3% hydrogen peroxide disinfectant (LIRCON, Cat#LEK-30100) and incubated away from light, then repaired with citrate buffer or EDTA under high pressure. Serum sealing was performed after PBS cleaning. Next, 50 μL primary antibody working solution (ATP4A: MBL, D031-3, 1:300; Mist1: CST, 14896S, 1:40; LCN2: Abcam, ab125075, 1:200; LCN2: Abcam, ab216462, 1:2000; Ki67: Abcam, ab16667, 1:200) in tissues and incubated at 37 °C for 1 h. After immersion and cleaning in PBS, 50 μL of secondary antibody (ZSGB-BIO, Cat#2313D1015) was added to the tissue, incubated at room temperature for 30 min, then 50 μL of the color-developing agent DAB was added and developed under light microscope. All images were captured and photographed using a light microscope (Nikon E600).

### Immunofluorescence (IF)

For tissue, frozen sections were re-warmed at room temperature, serum was sealed for 30 min after rinsing with PBS, and the primary antibody working solution (GIF: Santa Cruz, SC-514523, 1:200) and (GSII: ThermoFisher, L21415, 1: 100), which was incubated at 37 °C for 2 h. The samples were then rinsed with PBS three times, and then the secondary antibody working solution (ZSGB-BIO, Cat#ZF-0313) was added, incubated at room temperature without light for 30 min, then rinsed with PBS for three times, and added with Hoechst 33342(Solarbio, Cat#C0031) to re-stain the nucleus for 5 min. For cells, 200 μL 4% paraformaldehyde was added to each well for 20 min, and after washing with PBS, 200 μL 1% Triton X-100 (Solarbio, Cat#T8200) was added to permeate for 20 min, then 200 μL 5% goat serum was added, sealed at room temperature for 1 h, and primary antibody working solution (MUC2: Proteintech, 27675-1-AP, 1:1000) was added before incubating the specimens overnight at 4 °C. After washing with PBS, fluorescent secondary antibody (ZSGB-BIO, Cat#ZF-0316) was added and incubated at room temperature for 1 h, followed by Hoechst 33342 staining of the nucleus for 10 min. The cells were observed and photographed under a fluorescence microscope (ZEISS, LSM 900).

### RNA sequencing

Standard extraction methods were used to extract RNA from mouse tissues, and strict quality control was carried out on RNA samples using an Agilent 2100 bioanalyzer (Agilent Technologies, CA, USA). The mRNA with a polyA tail was enriched with oligo (dT) magnetic beads and then randomly interrupted by divalent cations in the Fragmentation Buffer. Using fragmented mRNA as a template and random oligonucleotides as primers, the first cDNA strand was synthesized in the M-MuLV reverse transcriptase system, the RNA strand was degraded by RNase H, and the second cDNA strand was synthesized in a DNA polymerase I system using dNTPs as the raw material. The purified double-stranded cDNA was end-repaired, an A-tail was added, and the sequencing joints were connected. A cDNA of approximately 370-420 bp was screened using AMPure XP beads for PCR amplification and the PCR products were purified using AMPure XP beads. Finally, a library was obtained. After a qualified library check, different libraries were sequenced using Illumina NovaSeq 6000 after pooling according to the requirements of effective concentration and target data volume, and 150-bp paired end readings were generated. After quality control of the raw data, we used HISAT2 Build an index of the reference genome (v2.0.5) and cleaned the paired ends using HISAT2 (v2.0.5), and reads were compared with the reference genome. The feature counts (1.5.0-p3) were used to calculate the reads mapped to each gene, the FPKM of each gene based on the length of the gene, and the reading mapped to that gene. The edgeR software package (v.3.22.5) was used for differential expression analysis and the ClusterProfiler software (v.3.8.1) was used for enrichment analysis of the GO and KEGG pathways of differentially expressed genes.

### Reverse transcription-quantitative PCR(RT-qPCR)

Total RNA was extracted using TRIzol reagent. Briefly, the RNA was extracted using chloroform and precipitated using isopropyl alcohol. RNA samples were resuspended in DEPC-treated H_2_O, followed by reverse transcription into cDNA using FastKing gDNA Dispelling RT SuperMix (TIANGEN, KR118-02). Reverse transcription-quantitative PCR (RT-qPCR) was performed on a CFX Connect™ RealTime System (Bio-Rad, USA) using SuperReal PreMix Plus (TIANGEN, FP205-02) and specific primers. The relative expression levels of target genes were normalized to that of β-actin and calculated using the 2-^ΔΔCT^. Primers used in this experiment are as follows: β-actin (human), forward: 5′-CACCATTGGCAATGAGCGGTTC-3′, reverse: 5′-AGGTCTTTGCGGATGTCCACGT-3′; β-actin (mouse), forward: 5′-GGCTGTATTCCCCTCCATCG-3′, reverse: 5′-CCAGTTGGTAACAATGCCATGT-3′; CDX2, forward: 5′-GACGTGAGCATGTACCCTAGC-3′, reverse: 5′-GCGTAGCCATTCCAGTCCT-3′; KLF4, forward: 5′-CAGCTTCACCTATCCGATCCG-3′, reverse: 5′-GACTCCCTGCCATAGAGGAGG-3′; LCN2, forward: 5′-GGGAAATATGCACAGGTATCCTC-3′, reverse: 5′-CATGGCGAACTGGTTGTAGTC-3′.

### Western blotting

Cells and tissue were lysed in Lysis buffer (Solarbio, Cat#R0100) and RIPA buffer (Solarbio, Cat#R0010) containing protease inhibitors on ice for 30 min, followed by centrifugation at 12,000 rpm for 10 min at 4°C. Protein concentrations in the supernatants were measured using a BCA protein assay kit (Thermo Scientific, Cat#23225), and proteins were incubated at 100°C for 10 min. The PAGE Gel Fast Preparation Kit(BIOMAN, BIO9923) was used to prepare the glue, and samples were loaded at 30 μg protein per well, at a constant pressure of 80 V, and then transferred to 120 V electrophoresis layer glue, which was placed in the semi-dry box in the order of filter paper, film, glue, and filter paper; the film transfer time was 2 h. The film was exposed to 5% skim milk powder at room temperature for 1 h; PBST cleaned the sealing liquid once, and then the primary antibodies (STAT3: Abcam, ab68153, 1:1000; p-STAT3 (Y705): Abcam, ab76315, 1:2000; LCN2: Abcam, ab216462, 1:1000; β-actin: CST, 3700S, 1:1000) were added and incubated overnight in a shake bed at 4 ℃. PBST solution was added with the secondary antibody (CST, 7074S, 1:3000) and incubated in a room temperature shaker for 1 h, protein bands were detected by Touch Imager (E-BLOT, Touch Imager Pro).

### Analysis of published single-cell RNA data

In this study, we used Seurat v4 to analyze single-cell RNA datasets GSE134520^20^ and GSE150290^21^ using routine methods. Quality control, normalization, scaling, identification of variable genes, and clustering were performed. Cell groups were identified based on the expression of conventional marker genes **(**Table S3**)**. Differential expression analysis was performed using the "FindMarkers" function with default parameters.

### Multiplex Immunohistochemistry staining and analysis

For multiplex immunohistochemistry (mIHC) staining, the paraffin sections were soaked in xylene, anhydrous ethanol, 95% ethanol, 70% ethanol, pure water, and 10% neutral formaldehyde at 37 ℃ overnight. After washing with TBST, the sections were placed in a citric acid solution for thermal repair. Serum and seal were added at room temperature and the primary antibody was added and incubated at room temperature for 1 h. After cleaning with TBST, the secondary antibody working liquid was added and the membrane was incubated at room temperature for 10 min. After washing with TBST, the dye was added and the membrane was incubated at room temperature for 10 min. Subsequently, the steps of heat repair, serum blocking, and addition of the primary antibody, secondary antibody, and dye were repeated. The following primary antibodies were used: Mist1 (CST, 14896S, 1:40), MUC6 (Santa Cruz, SC-33668, 1:200), MUC5AC (Invitrogen, YB372388, 1:200), and LCN2 (Abcam, ab125075, 1:200). Dye (Absin, abs50014) drops were added in the following order: 650, 520, 570, and 620. Next, DAPI working liquid was added, glass was covered, and images were collected using a multispectral tissue imaging analyzer (Akoya Biosciences, Phenoimager).

### Profiling of Lut-interacting proteins in cell lysate by photo-affinity chemoproteomics

GES-1 cells were cultured in a 10-cm dish. When cell density reached 90%, cells were washed twice with PBS and transferred to a 1.5 mL centrifuge tube. The cells were suspended with 0.1% Triton-PBS, ultrasonically split and centrifuged at 2000 ×*g* at 4℃ for 20 min. The supernatant was collected, and the concentration of the supernatant protein was quantified using BCA. The protein concentration was normalized to 2 mg/mL. A total of 3 groups were set up (i.e., “-UV group” without UV lights with the probe, the “+UV group” with the probe for UV lights, and the “competitive group” with Lut and probe for UV crosslinking. The lysate was precipitated using methanol-chloroform and washed three times with cold methanol. The proteins were resuspended in 0.4% sodium dodecyl sulfate (SDS)-PBS. The proteomes were conjugated with a biotin tag for enrichment using click chemistry. The proteomes were precipitated using methanol-chloroform, washed three times with cold methanol, and resuspended in 1.2% SDS-PBS. The proteins were incubated with streptavidin beads at 29 degree for 4 h. The beads were then then washed with PBS for 3 times and H_2_O for 3 times, denatured in 6 M urea/TEAB, reduced with 10 mM DTT for 30 min, alkylated with 20 mM IAA for 30 min and digested with trypsin (0.5 μg/μL) for 16 h. The digested mixtures were sequentially dimethylated with light, medium, and heavy tags, using different combinations of formaldehyde and sodium cyanoborohydride. LC-MS/MS was performed using a Q-Exactive Orbitrap mass spectrometer and an Ultimate 3000 LC system. The mobile phase A and B buffers of the HPLC system were 0.1% formic acid in H_2_O and 0.1% formic acid and 80% acetonitrile in H_2_O, respectively. The mass spectrometry conditions were as follows: In positive-ion mode, full-scan mass spectra were acquired in the m/z range of 350-1800 using an Orbitrap mass analyzer with a resolution of 70000. MS/MS fragmentation was performed in a data-dependent mode, in which the top 20 most intense ions were selected for MS/MS analysis. LC-MS/MS data were analyzed using ProLuCID software. The isotopic modifications included 28.03130, 32.05641, and 36.07567 Da for light, medium, and heavy labeling, respectively, which were set as variable modifications. The results were filtered using DTASelect and the ratio of reductive dimethylation was quantified using CIMAGE software.

### Validation of the interaction of Lut and STAT3

STAT3 was cloned in the pcDNA3.1-3Xflag plasmid. The plasmid pcDNA3.1-STAT3-3Xflag plasmid was transfected into HEK293T cells using PEI for 48 h. Cells were collected, resuspended in 0.1% Triton-PBS, ultrasonically lysed, and centrifuged to obtain supernatant. The concentration of supernatant protein was quantified using BCA and normalized to 2 mg/mL. Similarly, we set three groups: -UV group, +UV group, and competitive group. Next, we taked 5ul of each group as the input. The lysate was precipitated using methanol chloroform and washed three times with cold methanol. Proteins were resuspended in 0.4% sodium dodecyl sulfate (SDS)-PBS. The proteins were conjugated to a biotin tag for enrichment using Click chemistry. Finally, the protein loading buffer was added and the enriched protein was analyzed by western blotting, which was used as the output for each group. As STAT3 has a Flag tag, the amount of STAT3 in each group before and after enrichment was evaluated using an anti-Flag antibody (Proteintech, 66008-4-1g).

Expression and purification of the STAT3 (127-722) protein: Complementary DNA fragments of the corresponding human STAT3 (127-722) protein were cloned into a pHis-SUMO vector. The clone was expressed in LB at 37 °C overnight in BL21(DE3) cells. The overnight culture was amplified in 1 L LB at 37 °C for 3 h. The culture was then induced with IPTG at 16 °C overnight. The cells were then harvested by centrifugation. The pellet was resuspended in lysis buffer (50 mM Tris [pH 8.5], 150 mM NaCl and 1 mM DTT). The lysate was cleared by centrifugation at 12000 rpm for 30 min. STAT3 (127-722) was purified from the lysate using Ni-NTA (YEASEN, 20504ES08) and cleaved overnight with a SUMO protease.

SPR: Biacore T200 was used to determine the Kd values for STAT3 (127-722) and Lut. A Series S Sensor Chip CM5 was used to fix STAT3 (127-722). Lut was diluted in PBSP buffer.

### Statistical analysis

SPSS21.0 statistical software was used to analyze the data. When the measurement data followed a normal distribution, the mean±standard error of the mean was used for statistical description, and a paired t test, an unpaired t test, and a one-way ANOVA were used. When the data did not follow a normal distribution or were classified data, the median(Q1, Q3) was used for the statistical description and the rank sum test was used, and *P*<0.05 was considered statistically significant.

## Results

### Lut effectively inhibited SPEM formation

**Figure 1:**
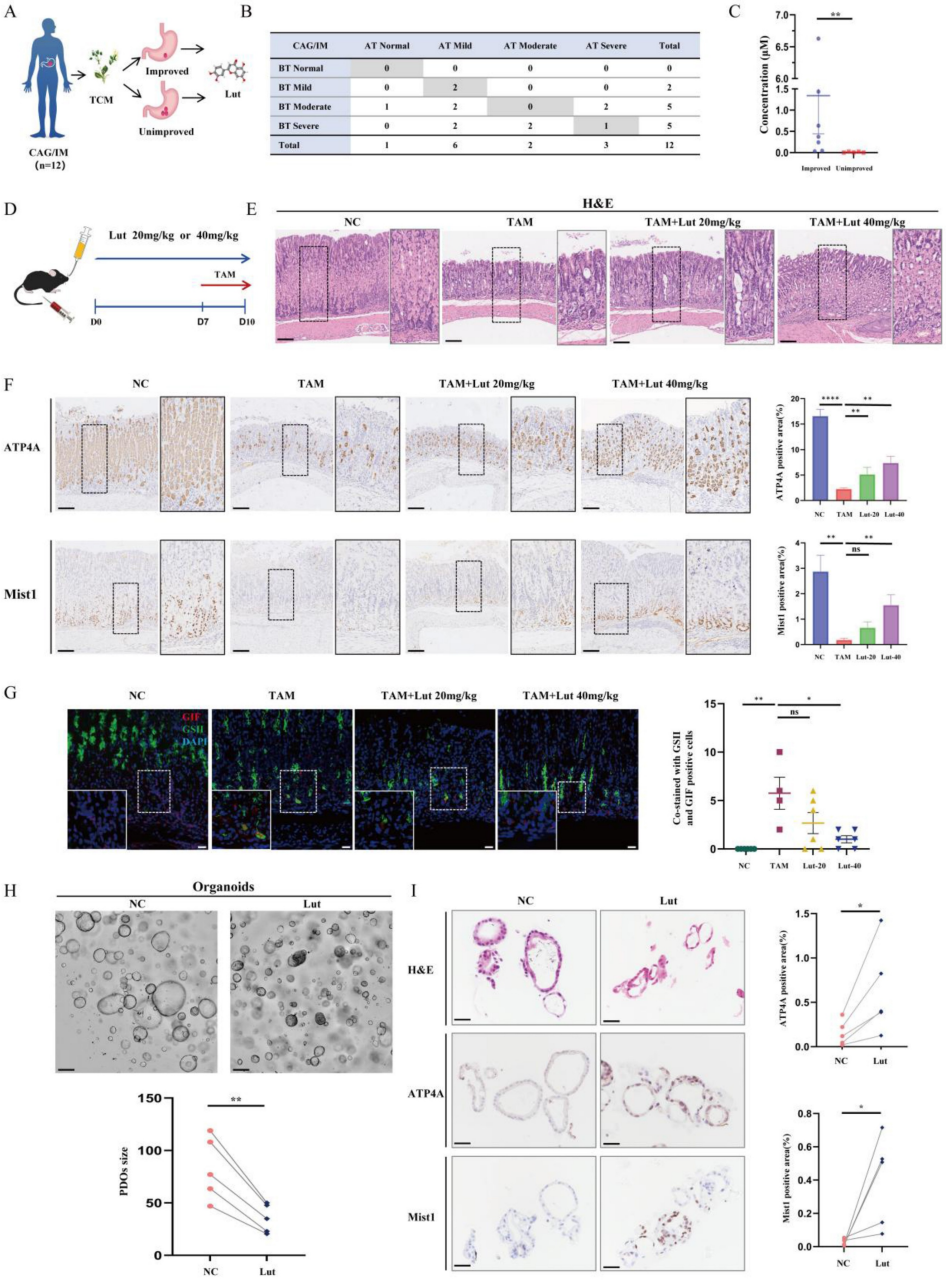
**Protective effect of Lut against CAG/IM patients and intervention of TAM.** (A) Schematic image showing detection of Lut in serum in TCM intervention patients. (B) Histopathology of the patient's gastric mucosa compartments during the follow-up period. (C) Comparison of serum Lut content in patients with and without pathological improvement. N=5-7. (D) Schematic image showing 20 or 40 mg/kg Lut in the treatment of TAM-intervention mice. (E) H&E images of the gastric corpus from TAM intervention mice treated with Lut. N=6. Scale bars: 100 μm. (F) Representative IHC images for ATP4A and Mist1 in the gastric corpus of mice. N=6. Scale bar: 100 μm. (G) Representative IF images of GSII and GIF double positive cells in the gastric corpus of mice. N=4-6. Scale bars: 50 μm. (H) Representative bright-field and sizes of organoids in Lut-treated and NC. N=5. Scale bars: 100 μm. Fifteen organoids per well were measured. (I) Representative H&E and IHC images for ATP4A and Mist1 of organoids treated with Lut and NC. N=5. Scale bars: 100 μm. All data are presented as mean±SEM.^ *^P<0.05, ^**^P<0.01, ^****^P<0.0001. CAG, chronic atrophic gastritis; IM, intestinal metaplasia; TCM, traditional Chinese medicine; Lut, luteolin; BT, before treatment; AT, after treatment; H&E, hematoxylin and eosin; NC, negative control; TAM, tamoxifen; IHC, immunohistochemistry; IF, immunofluorescence; ns, no statistical significance.

Using mass spectrometry, we found Lut was commonly expressed in two effective drugs for gastritis (Yinlian Hewei granules and Shiwei Baihe granules) (Figure S1). We further collected serum samples from 12 patients with chronic atrophic gastritis (CAG) or IM before and after treatment with traditional Chinese medicine (TCM) to detect serum Lut content (Figure 1A). Among them, seven patients had improved gastric mucosal pathology after TCM treatment, and the other five patients did not have improved gastric mucosa after treatment (Figure 1B). Notably, we found that the Lut content in patients with improved gastric mucosa was significantly higher than that in patients without improvement, indicating that Lut is an medicative ingredient that improves the precancerous conditions of the gastric mucosa* in vivo* (Figure 1C).

To study the function of Lut, we induced the acute SPEM model with the use of high-dose TAM (Figure 1D). As shown in Figure 1E, TAM-treated mice developed abnormal gland structures with apparent epithelial cell damage, while Lut-treated mice showed relatively intact gland structures, particularly those of the 40 mg/kg group. Further IHC results showed that the reduction in the number of ATP4A parietal cells and Mist1 chief cells was significantly alleviated in Lut-treated mice (Figure 1F). Finally, SPEM cells (both GSII^+^ and GIF^+^), which increased in mice treated with TAM, were also significantly reduced in Lut-treated mice (Figure 1G). These results suggested that Lut effectively inhibited TAM-induced SPEM formation.

To further explore the relationship between the effect of Lut and medication time, we treated mice with Lut for 4 and 10 days (Figure S2A). Consistently, TAM-induced mice developed abnormal gland structures, whereas mice treated with Lut for 10 days showed relatively intact gland structures, which were more effective than those treated with Lut for 4 days (Figure S2B). The IHC results showed that the reduction in the number of ATP4A parietal cells and Mist1 chief cells was significantly alleviated in mice treated with Lut for 10 days (Figure S2C). Furthermore, IF analysis showed that the number of SPEM cells was significantly reduced in 10 days-treated group (Figure S2D). Therefore, a longer medication time is beneficial to protect against gastric mucosal damage.

Subsequently, we examined the effects of Lut in human origin organoids with metaplasia of the pyloric gland. The growth of organoids was inhibited, and the volume of organoids decreased significantly after Lut treatment (Figure 1H). Consistently, the IHC results showed that the reduction in the number of ATP4A parietal cells and Mist1 chief cells were significantly alleviated in the Lut-treated group (Figure 1I). Thus, Lut effectively inhibits TAM-induced SPEM formation in both mouse models and human organoids.

To explore whether Lut has the drug adverse effects, we administered Lut to mice for 10 days (Figure S3A). The results did not show significant differences in liver function (ALT, *P*=0.26; AST, *P*=0.14) or renal function (BUN, *P*=0.18; Cr, *P*=0.53) compared with those in the NC group (Figure S3B). Histological results showed that there was no obvious damage to the liver, heart, spleen, kidney and intestinal tract after Lut intervention, proving the relative safety of Lut (Figure S3C).

### Lut effectively alleviated CDCA-induced gastric mucosal injury

**Figure 2:**
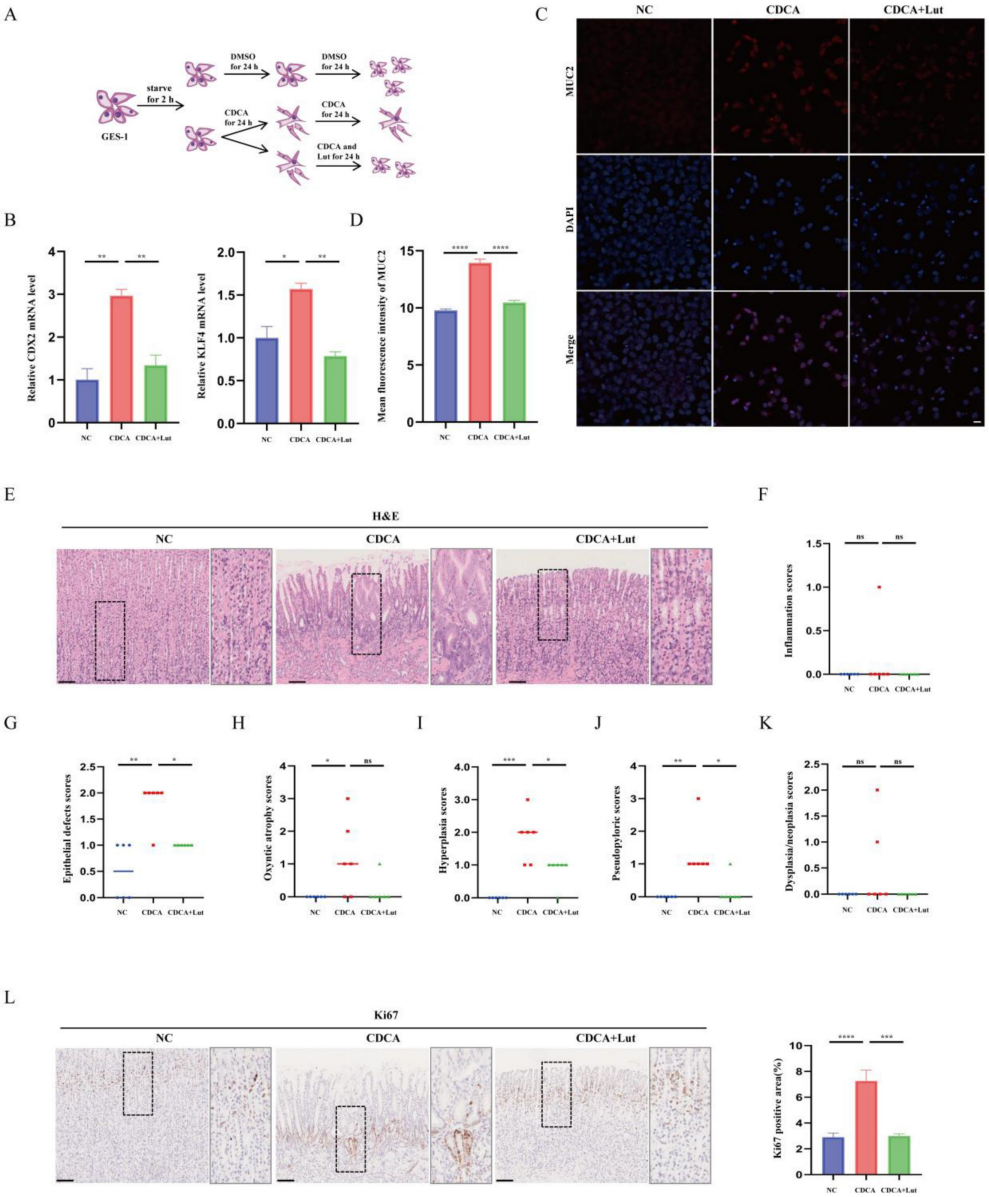
**Protective effect of Lut against CDCA-intervened models.** (A) Schematic image showing the CDCA intervention and Lut treatment for cells. (B) RT-qPCR for CDX2 and KLF4; the mRNA expression was normalized to β-actin. N=3. (C) Representative IF image for MUC2 in CDCA-intervened and Lut-treated cells. N=3. Scale bars: 20 μm. (D) The mean fluorescence intensity of MUC2. N=3. (E) H&E images of the stomach of CDCA-intervention rats treated with Lut. N=6. Scale bars: 100 μm. (F) Graph for inflammation scores of stomach of rats. N=6. (G) Epithelial defect scores in the stomach of rats. N=6. (H) Oxyntic atrophy scores of the stomach of rats. N=6. (I) Hyperplasia scores of the stomachs of rats. N=6. (J) Pseudopyloric scores of stomachs of rats. N=6. (K) Dysplasia/neoplasia scores of stomachs of rats. N=6. (L) Representative IHC images for Ki67 in stomach specimens of rats. N=6. Scale bar: 100 μm. Data are presented as mean±SEM or median (Q1, Q3). ^*^P<0.05, ^**^P<0.01, ^***^P<0.001, ^****^P<0.0001. DMSO, dimethyl sulfoxide; CDCA, chenodeoxycholic acid; IF, immunofluorescence; NC, negative control; Lut, luteolin; RT-qPCR, reverse transcription-quantitative PCR; H&E, hematoxylin and eosin; IHC, immunohistochemistry; ns, no statistical significance.

Atrophic gastritis, SPEM, and IM are all precancerous gastric conditions^22^. To further verify the effect of Lut under precancerous gastric conditions, we constructed a CDCA-treated cell model that mimicked IM^23^ (Figure 2A). As shown in Figure 2B, cells treated with CDCA exhibited increased IM-associated indices CDX2 and KLF4, while cells treated with Lut showed attenuated CDCA-induced upregulation of CDX2 and KLF4. Consistently, IF results showed increased IM-associated indices of MUC2 in CDCA-treated cells, whereas attenuated upregulation of MUC2 by CDCA was detected in cells treated with Lut (Figure 2C and 2D).

In addition, we established a chronic rat model of CDCA-induced gastric mucosal injury. Histological score results showed that CDCA-treated rats developed abnormal gland structures with significant epithelial damage, oxyntic atrophy, hyperplasia, and pseudopyloric metaplasia, whereas the Lut-treated rats showed obvious recovery of epithelial damage, hyperplasia, and pseudopyloric metaplasia except for oxyntic atrophy (Figure 2E, 2G-J). In particular, inflammation and dysplasia/neoplasia were rare in CDCA-induced rat models (Figure 2F and 2K). IHC analysis showed that the amount of Ki67 in gastric mucosal lesions increased significantly in CDCA-treated rats, while Lut-treated rats showed a significant decrease in the amount of Ki67 (Figure 2L). Therefore, in addition to SPEM, Lut has a protective effect on the CDCA-induced gastric mucosal injury.

### LCN2 was upregulated during pyloric gland metaplasia and decreased after Lut intervention

**Figure 3:**
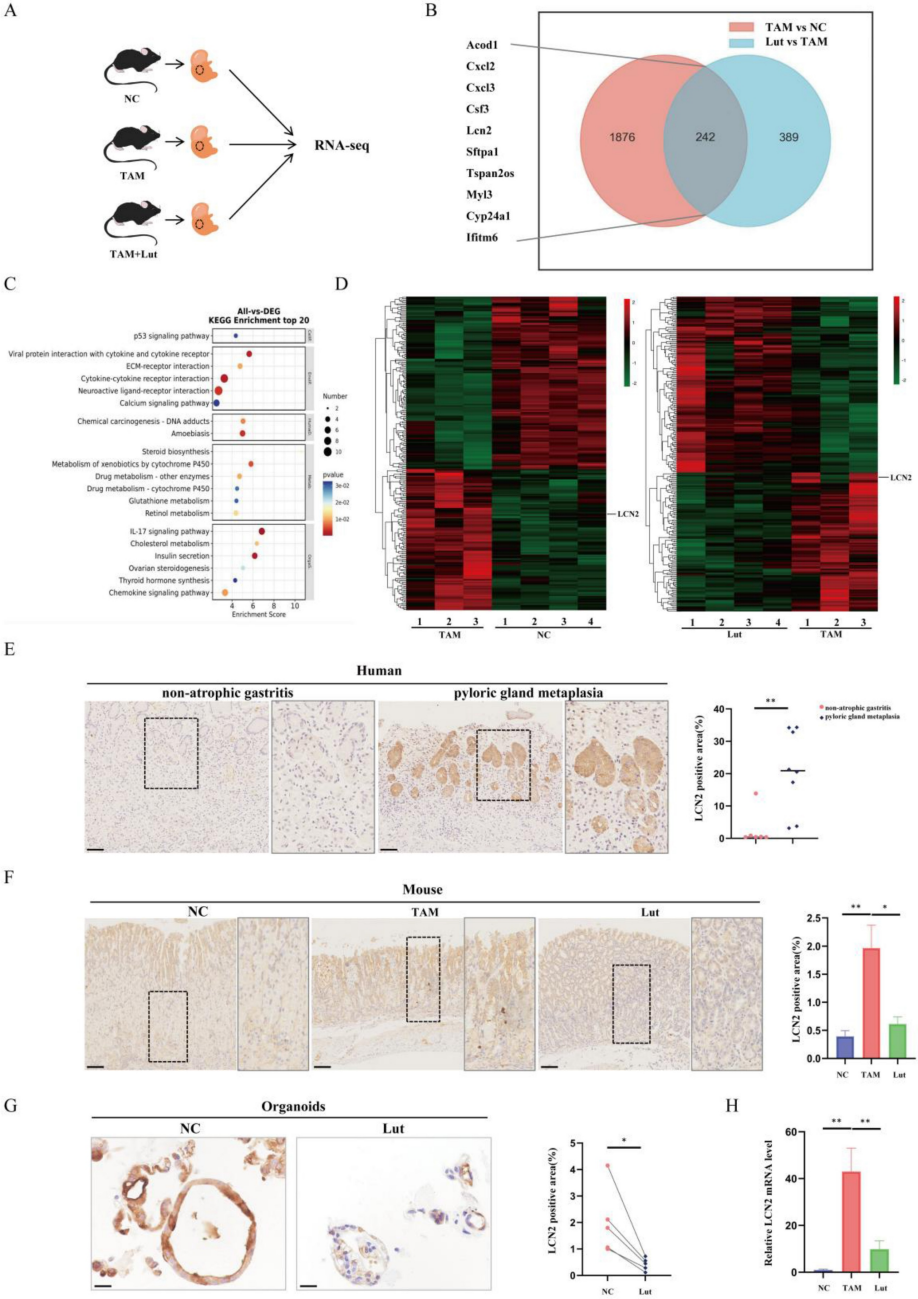
**RNA-seq of the gastric mucosa and LCN2 expression in humans, mice and organoids.** (A) Schematic image showing the RNA-seq strategy for the TAM-treated, Lut-treated, and NC mice. N=3-4. (B) The Venn diagram of 242 DEGs obtained by overlapping TAM vs. NC and Lut vs. TAM. (C) Bubble plot showing the top 20 selected KEGG enriched terms. (D) Heatmap showing DEGs in TAM-treated compared with NC mice and Lut-treated compared with TAM-treated mice. (E) Representative IHC images for LCN2 in non-atrophic gastritis and pyloric gland metaplasia of human tissue. N=6-8. Scale bar: 100 μm. (F) Representative IHC images for LCN2 in the gastric corpus of mice. N=8-9. Scale bar: 100 μm. (G) Representative IHC images for LCN2 in organoids. N=5. (H) RT-qPCR for LCN2 in stomach of mice; the mRNA expression level was normalized to β-actin. N=3-4. All data are presented as mean±SEM. ^*^P<0.05, ^**^P<0.01. RNA-seq, RNA sequencing; TAM, tamoxifen; Lut, luteolin; NC, negative control; DEGs, differentially expressed genes; KEGG, Kyoto Encyclopedia of Genes and Genomes pathways; IHC, immunohistochemistry; RT-qPCR, reverse transcription-quantitative PCR.

To further explore the mechanism of action of Lut on precancerous gastric conditions, RNA sequencing analysis was performed using samples from NC, TAM-induced and Lut-treated mice (Figure 3A). As shown in Figure 3B, significant differences were found in the transcriptomes of mice samples from NC and TAM-treated mice, as well as TAM-treated and Lut-treated mice, which shared 242 different genes. The KEGG pathway analysis revealed that immune-related pathways (IL-17 signaling pathway and cytokine-cytokine receptor interaction) were altered in mice treated with TAM and Lut (Figure 3C).

Notably, in the IL-17 signaling pathway, LCN2 expression levels were significantly increased in the acute SPEM model induced with TAM, but decreased in the Lut-treated mice (Figure 3D). IHC revealed a strong expression of LCN2 in the human gastric corpus with metaplasia of the pyloric gland, whereas the gastric corpus with non-AG showed minimal expression of LCN2 (Figure 3E). High LCN2 expression was also detected in the corpus glands of TAM-treated mice, while the corpus glands of Lut-treated mice showed decreased LCN2 expression (Figure 3F). Consistently, LCN2 expression decreased significantly in organoids after Lut treatment (Figure 3G). Additionally, overall LCN2 mRNA expression increased in the stomachs of TAM-treated mice but decreased in the stomachs of Lut-treated mice (Figure 3H).

### Blocking LCN2 suppressed TAM-induced oxyntic atrophy and metaplasia and partially counteracted the effect of Lut

**Figure 4:**
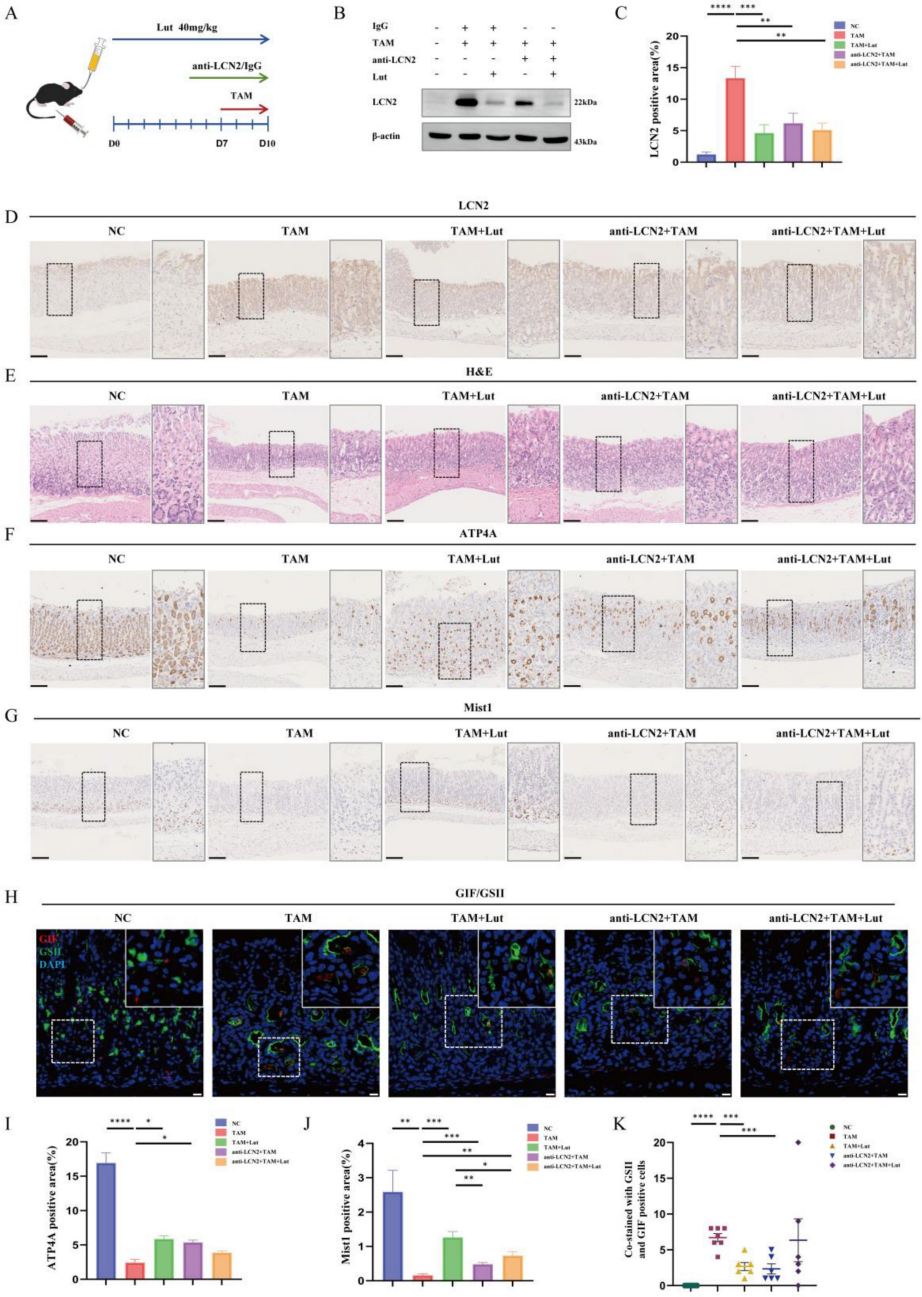
**Inhibition of oxyntic atrophy and metaplasia and counteraction the effect of Lut in LCN2 blocking mice after TAM intervention.** (A) Schematic image showing anti-LCN2 antibody and Lut for TAM-treated mice. (B) Western blotting for LCN2 in stomach of mice. (C) Graph of positive area of LCN2. N=6-8. (D) Representative IHC images for LCN2 in the gastric corpus of mice. N=6-8. Scale bar: 100 μm. (E) H&E images in the gastric corpus of mice. N=6-8. Scale bars: 100 μm. (F) Representative IHC images for ATP4A in the gastric corpus of mice. N=6-8. Scale bars: 100 μm. (G) Representative IHC images for Mist1 in the gastric corpus of mice. N=6-8. Scale bars: 100 μm. (H) Representative IF images of GSII and GIF double positive cells in the gastric corpus of mice. N=6-7. Scale bars: 50 μm. (I) Graph of positive area of ATP4A. N=6-8. (J) Graph of positive area of Mist1. N=6-8. (K) Co-stained with GSII and GIF positive cells. N=6-7. All data are presented as mean±SEM. ^*^P<0.05, ^**^P<0.01, ^***^P<0.001, ^****^P<0.0001. TAM, tamoxifen; Lut, luteolin; IHC, immunohistochemistry; NC, negative control; H&E, hematoxylin and eosin; IF, immunofluorescence.

LCN2 has previously been reported increasing significantly in the gastric mucosa of individuals with *H. pylori* infection but down-regulated in GC^14^,^24^. To further confirm the role of LCN2 in SPEM, we treated TAM-induced mice with an anti-LCN2 antibody to achieve a targeted blockade of LCN2 (Figure 4A). As shown in Figure 4B, we observed an expected reduction in the level of LCN2 protein in the stomach after administration of anti-LCN2 antibodies. Similarly, IHC showed that LCN2 levels in the gastric corpus were reduced upon administration of anti-LCN2 antibody (Figure 4C and 4D). Further histological results showed that anti-LCN2 antibody treatment alleviated gastric mucosal damage caused by TAM (Figure 4E), indicating that blocking LCN2 conferred protective effects in TAM-induced mice. IHC revealed that the reduction in the number of ATP4A parietal cells and Mist1 chief cells was significantly alleviated by anti-LCN2 antibody treatment (Figure 4F, 4G, 4I, and 4 J). IF showed that SPEM cells (both GSII^+^ and GIF^+^) were significantly reduced upon administration of the anti-LCN2 antibody (Figure 4H and 4K), indicating that the anti-LCN2 antibody had a protective effect on the stomachs of TAM-induced mice.

Next, we investigated whether the administration of anti-LCN2 antibodies influenced Lut efficacy. Compared with Lut treatment alone, combined factor intervention (Lut and anti-LCN2 antibody) did not further improve the degree of oxidative atrophy and metaplasia recovery. Although the reduction in the number of Mist1 chief cells was alleviated by Lut and anti-LCN2 antibody treatment (Figure 4G and 4J), the reduction in the number of ATP4A parietal cells was not alleviated (Figure 4F and 4I). SPEM cells were not reduced upon administration of Lut and anti-LCN2 antibodies (Figure 4H and 4K). Similarly, compared with blocking LCN2, combined factor intervention (Lut and anti-LCN2 antibody) also did not further enhance the degree of oxidative atrophy and metaplasia recovery. Recovery of ATP4A parietal cells and Mist1 chief cells and reduction of SPEM cells (both GSII^+^ and GIF^+^) were not significantly different between the two groups (Figure 4F-K), indicating that LCN2 is the dominant regulatory protein of Lut in TAM-induced mice.

### Lut could bind to STAT3 with high affinity

**Figure 5:**
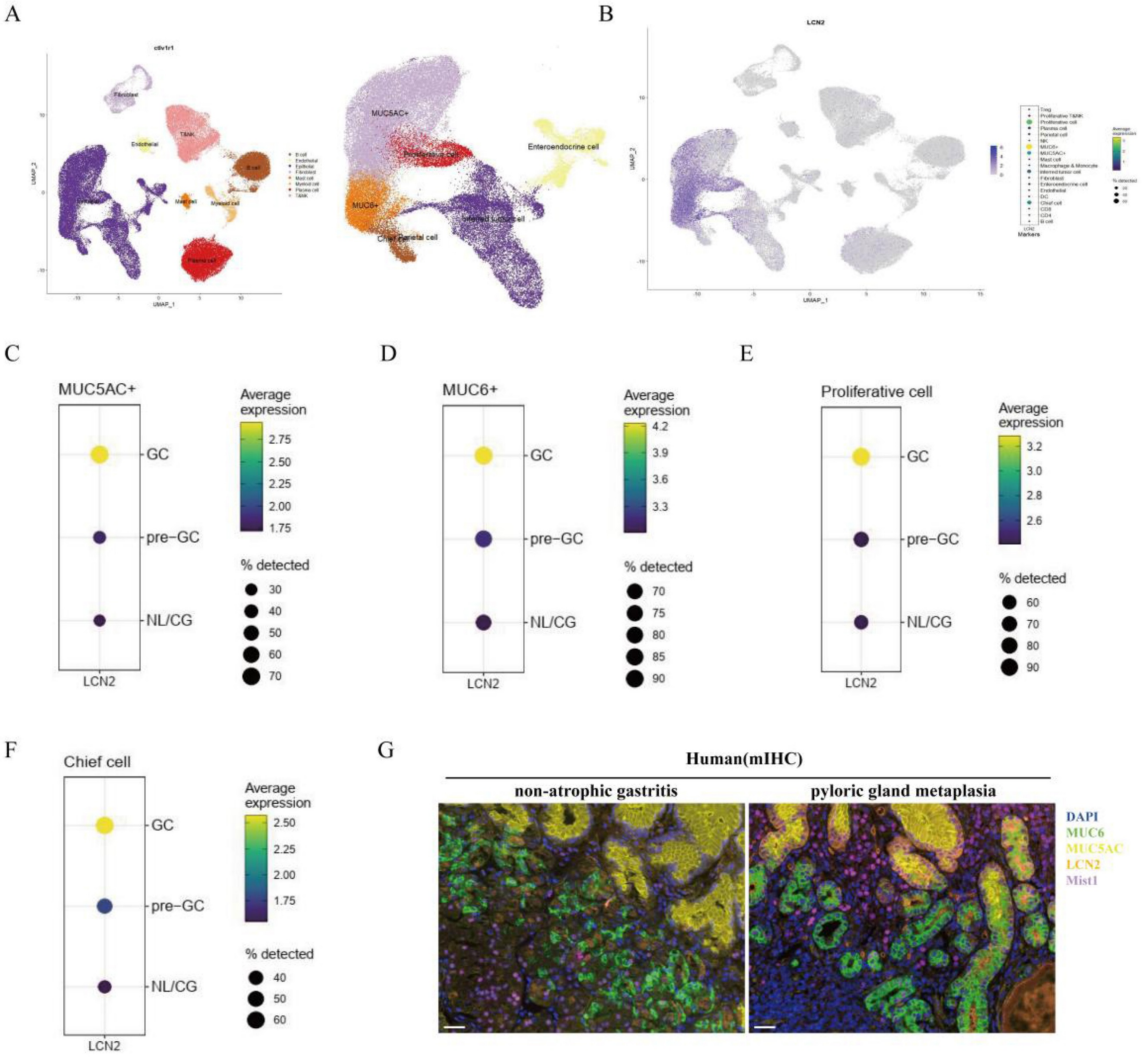
**Expression site and cell origin of LCN2.** (A) Identification of cell clusters in gastric tissue (normal mucosa/precancerous lesions/gastric adenocarcinoma). The whole cell data were subclustered into eight group using conserved maker genes. Epithelial cells were subclustered into six group using conserved maker genes. LCN2 expression in (B) gastric tissue, (C) MUC5AC^+^ epithelial cells in normal mucosa/precancerous lesions, and gastric adenocarcinoma groups, (D) in MUC6^+^ epithelial cells in normal mucosa/precancerous lesions, and gastric adenocarcinoma groups, (E) in proliferative epithelial cells in normal mucosa/precancerous lesions, and gastric adenocarcinoma groups, and (F) in chief cells in normal mucosa/precancerous lesions, and gastric adenocarcinoma groups. (G) Representative images of mIHC showing the LCN2 and composition of corpus cell lineage in human. LCN2 was labeled as orange; MUC6, MUC5AC, Mist1, and DAPI were labeled as mucous neck cells (green), foveolar cells (yellow), chief cells (purple), and nuclei (blue), respectively. Scale bar: 100 μm. mIHC, multiplex immunohistochemistry staining.

Previous single-cell RNA data showed that LCN2 is expressed mainly in epithelial cells, especially MUC6^+^, MUC5AC^+^, and proliferative cells (Figure 5A). In particular, LCN2 expression levels were higher in MUC6^+^, MUC5AC^+^, proliferation cells, and in the chief cells in pre-GC and GC than gastritis (Figure 5B-F). To further verify the cellular origin of LCN2, we performed mIHC, and the results showed that there were a large number of cells co-staining with MUC6 and LCN2, and some cells co-staining with MUC5AC and LCN2 in both the gastric corpus with pyloric gland metaplasia samples and non-atrophic gastritis, indicating that LCN2 is expressed primarily in epithelial cells, and the increased LCN2 during pyloric gland metaplasia mainly originates from epithelial cells (Figure 5G).

**Figure 6:**
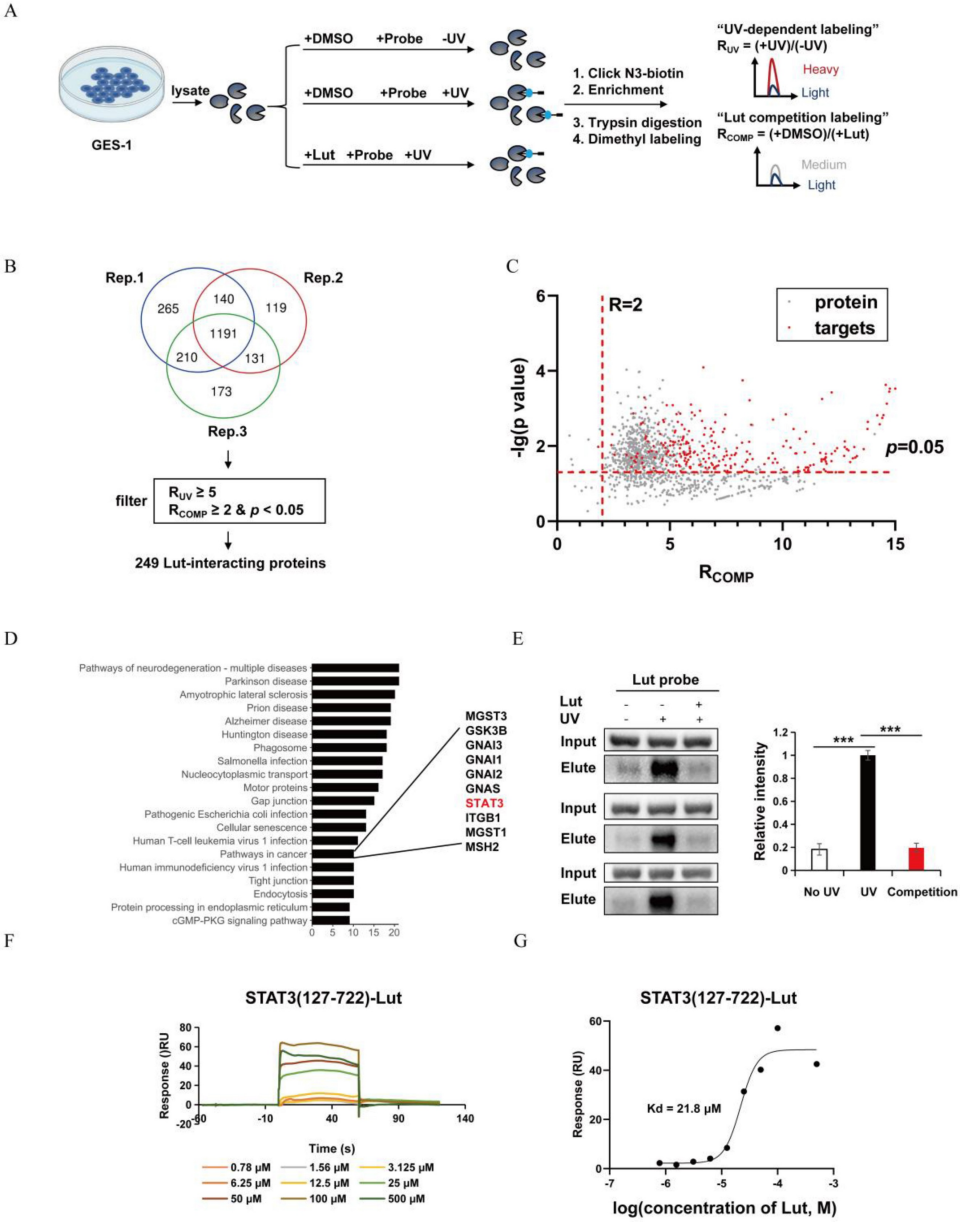
**Target profiling of Lut by quantitative chemoproteomics and direct interaction of STAT3 and Lut.** (A) Schematic image showing the workflow of profiling of Lut-interacting proteins by quantitative chemoproteomics. (B) Venn diagrams showing the number of identified Lut-interacting proteins from the lysates of GES-1 cells. (C) Ratio distribution of proteins enriched by the Lut photo-cross-linking probe. (D) KEGG analysis of the 249 protein targets identified by quantitative chemoproteomics. (E) Experimental validation of Lut-STAT3 interactions by western blotting. (F) The binding affinities between Lut and STAT3 was detected by SPR analysis. (G) The Kd value of Lut and STAT3 was determined as 21.8 μM by SPR. All data are presented as mean±SEM.^ ***^P<0.001. SPR, surface plasmon resonance.

To identify Lut targets, quantiative chemoproteomics was performed using a Lut probe (Figure 6A). Given that Lut does not contain any obvious reactive moiety that can covalently modify proteins, we designed and synthesized a photo-affinity Lut probe containing a diazirine group and an alkynyl reporter group. The synthetic route of the Lut probe was shown in Figure S4. When the Lut probe (200 μM) was used to label the proteome in an in-gel fluorescence assay, cotreatment of the native Lut (400 μM) was able to compete the probe labeling, supporting that the probe binds to similar targets as Lut does (Figure S5).

As shown in Figure 6B and C, 249 Lut interacting proteins were identified in GES-1 lysate. KEGG pathway analysis revealed that these 249 Lut-interacting proteins were involved in cancer pathways (Figure 6D). Of them, STAT3 was related to GC and precancerous gastric conditions and was also reported to regulate *Lcn2* expression. So, we hypothesized that Lut might function through STAT3. Therefore, we pulled down HEK-293T cells transfected with FLAG-labeled STAT3 plasmid using streptavidin after probe treatment and photo-cross-linking. Western blotting showed that the Lut probe could label STAT3 after UV irradiation, and the labeling was significantly competed by Lut (Figure 6E). We then purified STAT3 (127-722), an important domain for the function of STAT3, to investigate the affinity between STAT3 (127-722) and Lut. The SPR assay showed that Lut interacted with STAT3 (127-722) with high affinity (Kd=2.18 ×10^-5^ mol/L) (Figure 6F and 6G).

### Lut downregulated LCN2 by binding STAT3 to protect the gastric mucosa

**Figure 7:**
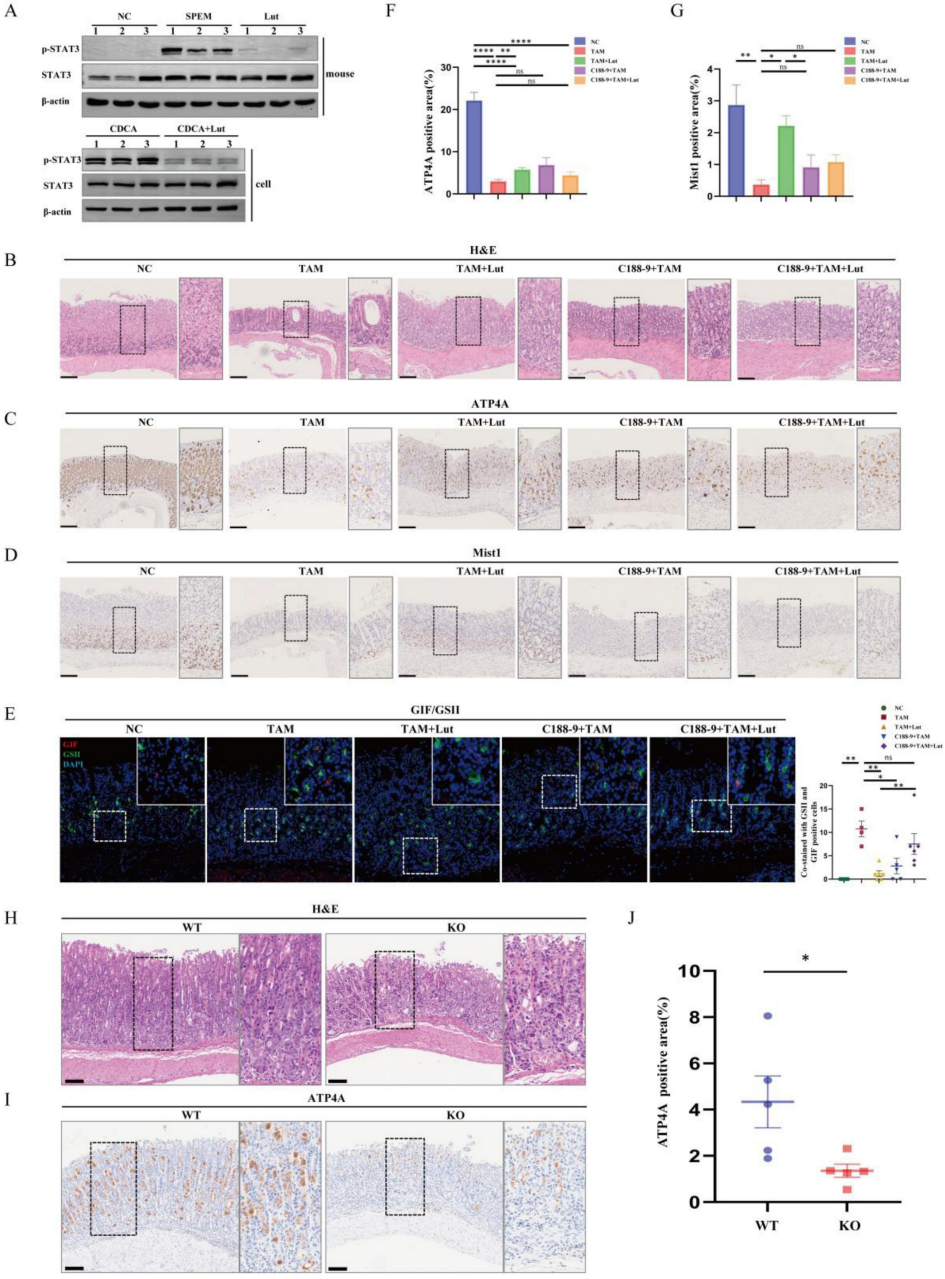
**Counteraction the effect of Lut in p-STAT3-inhibited mice and *Stat3^fl/fl^VillinCre* mice after TAM intervention.** (A) Western blotting for p-STAT3 and STAT3 in Lut-treated, TAM-treated mice, and CDCA-intervented cells. (B) H&E images in the gastric corpus of mice. N=4-7. Scale bars: 100 μm. (C) Representative IHC images for ATP4A in the gastric corpus of mice. N=4-7. Scale bars: 100 μm. (D) Representative IHC images for Mist1 in the gastric corpus of mice. N=4-7. Scale bars: 100 μm. (E) Representative IF images of GSII and GIF double positive cells in the gastric corpus of mice. N=4-6. Scale bars: 50 μm. (F) Graph of positive area of ATP4A. N=4-7. (G) Graph of positive area of Mist1. N=4-7. (H) H&E images of the corpus from WT(*Stat3^fl/fl^*) and KO(*Stat3^fl/fl^VillinCre*) mice during Lut treatment. Scale bars: 100 μm. (I) Representative IHC images for ATP4A in WT(*Stat3^fl/fl^*) and KO (*Stat3^fl/fl^VillinCre)* mice. N=5. Scale bar: 100 μm. (J) Graph of positive area of ATP4A. N=5. All data are presented as mean±SEM.^ *^P<0.05, ^**^P<0.01, ^***^P<0.001,^ ****^P<0.0001. Lut, luteolin; TAM, tamoxifen; CDCA, chenodeoxycholic acid; H&E, hematoxylin and eosin; IHC, immunohistochemistry; IF, immunofluorescence; WT, wild-type; KO, knockout.

Previous reports have indicated that p-STAT3 was highly expressed in gastric mucosa infected with *H. pylori,* and Lut could inhibit STAT3 phosphorylation, accelerate STAT3 degradation^11^,^25^, To detect the effects of Lut on STAT3 and p-STAT3 expression in gastric lesions, we performed protein detection and found that Lut could significantly downregulate the level of p-STAT3 but did not affect STAT3 expression in both TAM-induced mice and CDCA-treated cells (Figure 7A). Next, we explored whether inhibition of p-STAT3 with C188-9 had a protective effect in TAM-induced mice (Figure S6A). After the intervention of C188-9, we observed an expected reduction in the level of p-STAT3 in both mice and cells (Figure S6B). Surprisingly, there was no remission of TAM-induced oxidative atrophy after inhibition of p-STAT3, including a reduction in the number of ATP4A parietal cells and Mist1 chief cells, which was not significantly alleviated by C188-9 treatment (Figure 7B, 7C, 7D, 7F, and 7G), but SPEM cells (both GSII^+^ and GIF^+^) were reduced (Figure 7E), indicating that p-STAT3 may have played only a partial role in the development of SPEM.

Next, we investigated whether treatment with C188-9 affected the efficacy of Lut. Compared with Lut treatment alone, combined intervention (Lut and C188-9) did not further enhance the degree of oxidative atrophy and metaplasia recovery. IHC showed that the reduction in the number of ATP4A parietal cells and Mist1 chief cells was not alleviated by Lut and C188-9 (Figure 7C, 7D, 7F, and 7G). Notably, SPEM cells (both GSII^+^ and GIF^+^) were not reduced after treatment with Lut and C188-9 (Figure 7E). In addition, the same result was found in the corpus samples from Lut-treated *Stat3^fl/fl^* and epithelial cell-specific *Stat3* deficient (Villin-cre-*Stat3*^flox/flox^) mice with TAM treated (Figure 7H, 7I, and 7J). The gastric mucosa was severely damaged and the number of ATP4A parietal cells were remarkably reduced in Lut-treated Villin-cre-*Stat3*^flox/flox^ mice, indicating that STAT3 is an important target protein of Lut in mice induced by TAM. Furthermore, the overall expression of p-STAT3 in the stomach of mice was consistent with that of LCN2, which was upregulated in the gastric tissue of SPEM mice and downregulated after Lut treatment (Figure 4B and Figure 7A). Therefore, Lut may target STAT3 and down-regulate LCN2 to inhibit precancerous gastric conditions.

## Discussion

The multiple stages of precancerous conditions in GC include oxidative atrophy, SPEM, IM, and dysplasia. IM may develop from the existing SPEM, and the observation of SPEM cells in the deep glands with IM lineages in the glandular cavity may indicate the transformation of SPEM into IM or secondary differentiation^26^. To date, clinical guidance for best practices related to the management of precancerous gastric conditions remains limited, especially due to the lack of effective therapeutic drugs^27^. Natural products have received increasing attention from researchers worldwide and a growing number of natural products have been approved for cancer clinical studies^28^. In the present study, we found Lut was commonly enriched in two effective drugs for gastritis using mass spectrometry. Moreover, Lut content was higher in the serum of Chinese medicine treated patients with pathological improvement, suggesting that Lut content may be beneficial for pathological improvement. We further used a traditional acute model of high-dose TAM-induced oxidative atrophy and SPEM^29^, and a chronic model of gastric mucosal injury and IM induced by bile acids^30^ to investigate the effects of Lut on gastric mucosal injury. The results showed that Lut effectively inhibited TAM-induced oxidative atrophy and SPEM, and the effect of the high dose was more obvious than that of the low dose. Lut can recover epithelial damage, hyperplasia, and pseudopyloric metaplasia in a CDCA-induced model. However, we did not observe significant inflammatory infiltration or dysplasia/neoplasia, which may be due to the 6-month modeling time, which usually requires 12 months to induce severe gastric mucosal lesions^31^. Furthermore, we verified the function of Lut using organoids from pyloric gland metaplasia cultures for the first time.

The various functions of LCN2 have been explored, such as transporting hydrophobic ligands across cell membranes, regulating immune responses, maintaining iron balance, and promoting epithelial cell differentiation. It is upregulated in a variety of human diseases and cancers; for example, high levels of LCN2 are associated with breast, pancreatic, thyroid, ovarian, and colon cancer^32^,^33^. In this study, we also identified strong LCN2 staining in gastric mucosal tissue from pyloric metaplasia, and increased LCN2 levels during pyloric gland metaplasia originated mainly in epithelial cells, which is consistent with our analysis of the public databases. In addition, blocking LCN2 suppressed oxyntic atrophy, and SPEM in a TAM-induced model, including parietal cells, chief cells and SPEM cells, recovered to varying degrees, indicating that LCN2 may be a key factor in the development of SPEM. Previous results have shown that quercetin reduced the expression of LCN2 in *H.pylori* infected GES-1 cells, whereas strong expression of LCN2 eliminated the protective effect of quercetin on *H. pylori* infected GES-1 cells^34^. However, LCN2 as an effector of Lut has not been reported in gastric diseases. In the present study, we found that Lut could downregulate LCN2 expression, and combined factor intervention (Lut and anti-LCN2 antibody) did not further enhance the degree of oxidative atrophy and metaplasia recovery compared to Lut treatment alone, suggesting that Lut may be an important molecule regulated by Lut in gastric mucosal disease. The role of LCN2 in SPEM requires further study using multiple models and methods, such as LCN2 knockout mice.

Lut treatment can significantly inhibit STAT3 phosphorylation in SGC7901/DDP cells^25^. SPR indicated that Lut had a strong binding affinity for Src, an upstream STAT3 kinase^35^. Inhibition of cell cycle progression, colony formation, proliferation, migration, invasion, and selective killing of STAT3-overactivated GC cells have been observed in various cancer cells treated with Lut^25^,^36^. In our study, we further explored the direct Lut target in a CDCA-induced cell model using quantitative chemorpteomics. The results showed that STAT3 was a Lut direct acting protein and demonstrated that Lut could inhibit STAT3 phosphorylation in both the TAM-induced mouse model and the CDCA-induced cell model. We provided direct evidence for binding of Lut to STAT3.

STAT3 plays an important role in gastric mucosal diseases by regulating the expression of target genes in many solid tumors and being involved in a wide range of pro-inflammatory cancer cell processes^37^. Hyperactivation of STAT3 and p-STAT3 expression occurs in DCA-induced models, dysplastic organoids, GC cells, and tissues^11^,^38^,^39^. *H. Pylori*-infected C57BL/6 mice also developed SPEM 6 months after infection, accompanied by increased expression of IL-6, p-STAT3, and the proliferative marker Ki67^11^,^40^. In addition, inhibition of STAT3 by TTI-101 can have anticancer effects and effectively prevent the transformation of metaplasia into atypical hyperplasia^41^. In the present study, we found that p-STAT3 expression was elevated in TAM-treated mice, and the inhibition of STAT3 phosphorylation partially counteracted the effects of Lut in TAM-treated mice, indicating that STAT3 is an important target protein of Lut in SPEM. However, TAM-induced oxidative atrophy did not improve significantly after treatment with C188-9. This may be due to the limited *in vivo* stability of the inhibitor, and only part of STAT3 function can be inhibited by one inhibitor, such as blocking STAT3 phosphorylation, acetylation, dimerization, nuclear translocation, and STAT3 DNA binding activity. Thus, the role of STAT3 in oxidative atrophy and metaplasia requires further evaluation in STAT3 knockout mice, and the regulatory mechanism of Lut in STAT3 needs to be clarified.

LCN2 promotes ferroptosis from hypoxic-ischemic brain injury by activating the NF-κB/STAT3 signaling pathway^42^. Continuous activation of STAT3 in the spinal dorsal horn of a mouse model of dermatitis leads to upregulation of LCN2, which in turn leads to chronic pruritus^43^. In alcoholic liver injury, STAT3 activation triggers the secretion of downstream inflammatory factors, including LCN2^44^. Furthermore, a chromatin immunoprecipitation (ChIP) assay revealed that STAT3 was recruited to the promoter region of the LCN2 gene upon activation of STAT3 by IL-6^15^. Thus, LCN2 is an effector molecule regulated by STAT3 in many diseases. In turn, LCN2 can activate the STAT3 signaling pathway, forming bidirectional regulation, and affecting the disease process. In our study, we observed consistent expression levels of p-STAT3 and LCN2. Therefore, Lut may target STAT3 and down-regulate LCN2 to inhibit precancerous gastric conditions. However, the regulatory mechanisms between the two need to be further confirmed.

Collectively, our results emphasized the protective role of Lut in oxidative atrophy and metaplasia. We identified LCN2 as a new therapeutic target for precancerous gastric conditions, and demonstrated that STAT3 was a direct target of Lut. Lut could inhibit the STAT3/LCN2 axis by binding to STAT3 and inhibiting its phosphorylation. We believe our study may shed light on the role of the STAT3/LCN2 axis in precancerous conditions of the gastric mucosa and reveal new mechanisms of action of Lut. These findings revealed a novel strategy to inhibit gastric precancerous conditions.

## Supplementary Material

Supplementary figures and tables.

## Abbreviations

GC: gastric cancer
AG: atrophic gastritis
SPEM: spasmolytic polypeptide-expressing metaplasia
IM: intestinal metaplasia
Lut: luteolin
*H. pylori*: *Helicobacter pylori*
LCN2: lipocalin 2
TAM: tamoxifen
CDCA: chenodeoxycholic acid
NC: negative control
H&E: hematoxylin and eosin
IHC: immunohistochemical staining
IF: immunofluorescence
RT-qPCR: reverse transcription-quantitative PCR
mIHC: multiplex immunohistochemistry
CAG: chronic atrophic gastritis
TCM: traditional Chinese medicine
ChIP: chromatin immunoprecipitation
