# Supplementary material for: Luteolin improves precancerous conditions of the gastric mucosa by binding STAT3 and inhibiting LCN2 expression
Source: Int J Biol Sci. 2025 May 7;21(8):3397–415. doi: 10.7150/ijbs.111636 (PMC12160510; doi:10.7150/ijbs.111636)
Supplement: Supplementary file 1 — Supplementary figures and tables. [file ijbsv21p3397s1.pdf]

Supplementary materials

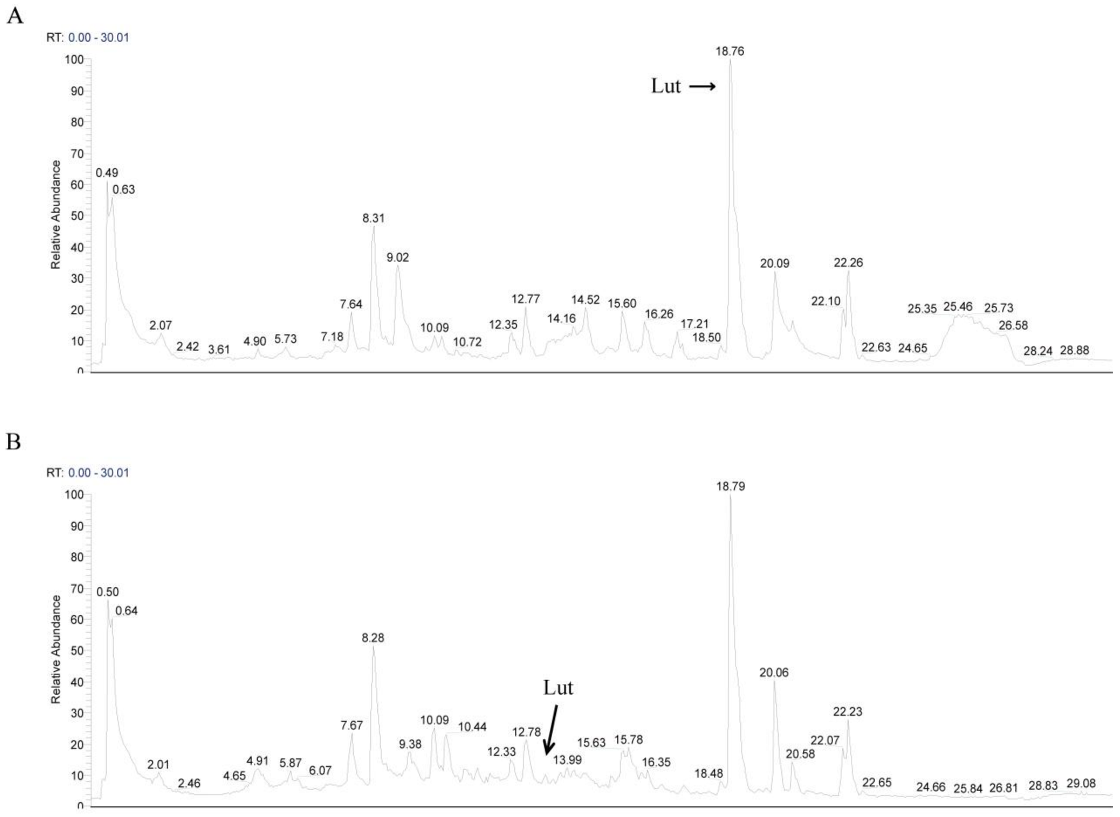

Figure S1. TIC in negative ionization mode. (A) TIC in negative ionization mode of Yinglian Hewei granules. (B) TIC in the negative ionization mode of Shiwei Baihe granules. TIC, total ion chromatogram.

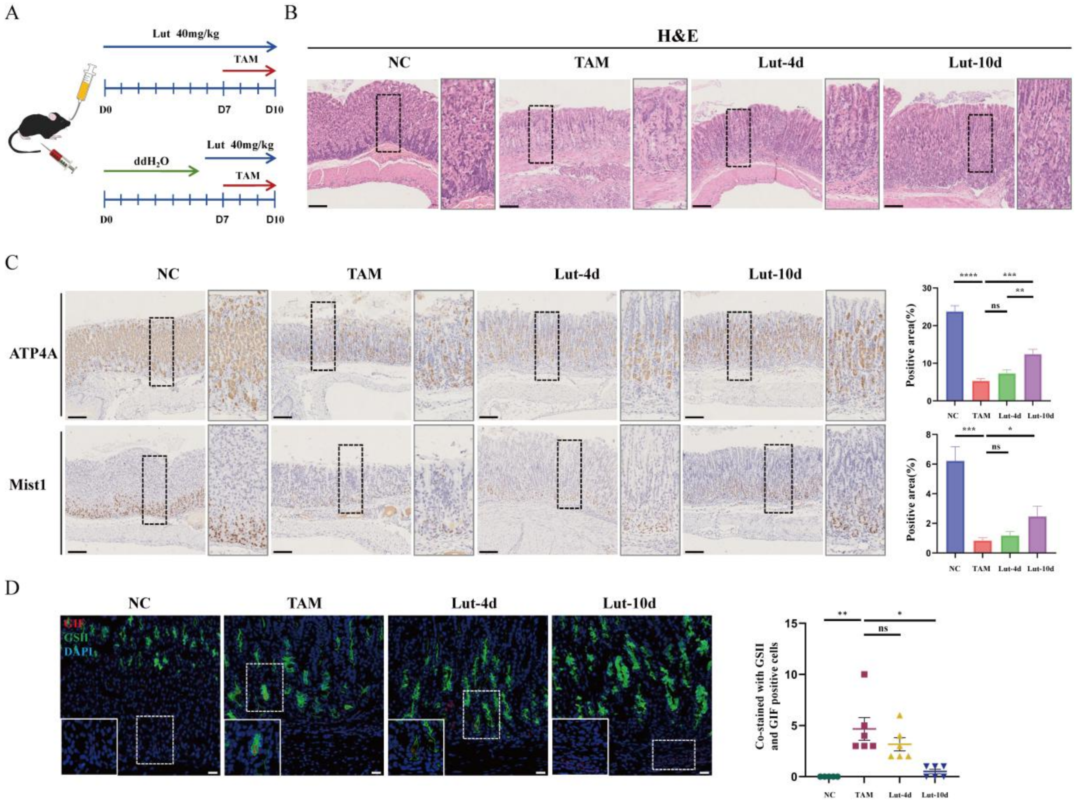

Figure S2. Protective effect of Lut against time-dependent TAM intervention. (A) Schematic image showing Lut for 4 or 10 days treatment of TAM intervention mice. (B) H&E images in the gastric corpus of mice. N=6. Scale bars: 100 μm. (C) Representative IHC images of ATP4A and Mist1 in the gastric corpus of mice. N=6. Scale bar: 100 μm. (D) Representative IF images of GSII and GIF double positive cells in the gastric corpus of mice. N=5-6. Scale bars: 50 μm. All data are presented as mean±SEM. $^{*}P$<0.05, $^{**}P$<0.01, $^{***}P$<0.001, $^{****}P$<0.0001. H&E, hematoxylin and eosin; NC, negative control; TAM, tamoxifen; Lut, luteolin; IHC, immunohistochemistry; IF, immunofluorescence; ns, no statistical significance.

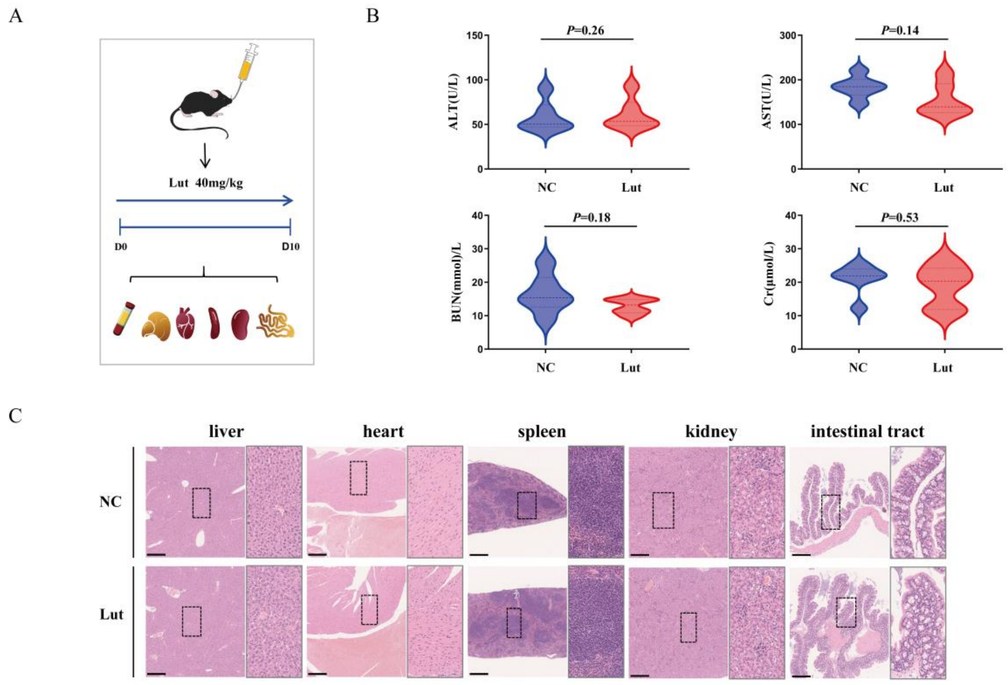

Figure S3. Safety of Lut. (A) Schematic image showing Lut intervention mice for 10 days. (B) Comparison of liver function (ALT and AST) and renal function (BUN and Cr) during Lut treatment and NC mice. N=6. (C) H&E images of liver, heart, spleen, kidney and intestinal tract from Lut treatment and NC mice. Scale bars: 100 μm. Data are presented as mean±SEM. H&E, hematoxylin and eosin; Lut, luteolin; NC, negative control.

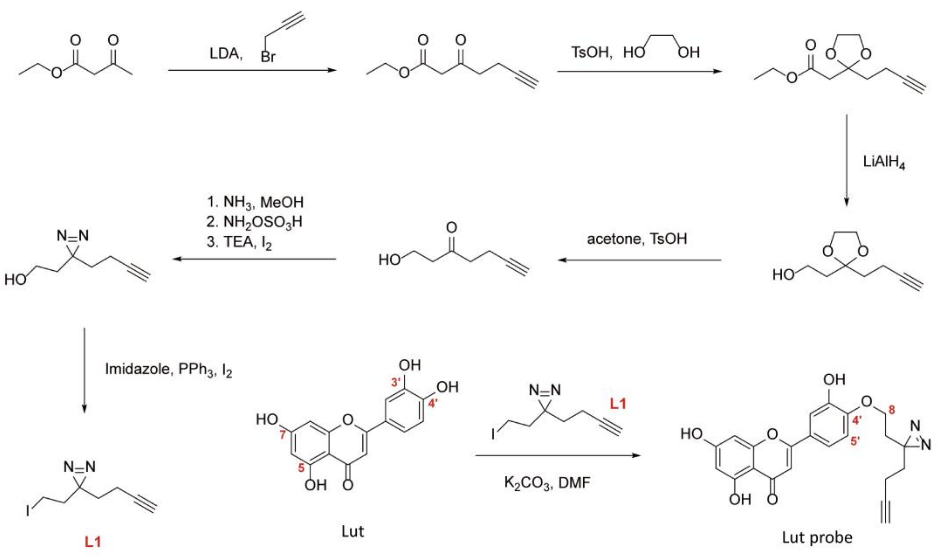

Figure S4. The synthetic route of the Lut probe. Lut, luteolin.

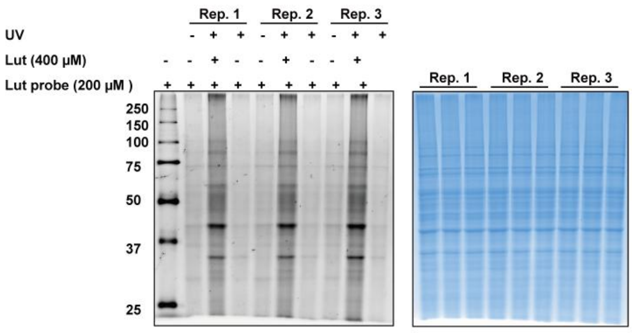

Figure S5. Evaluation of the labeling efficiency of the Lut probe based on in-gel fluorescence. Lut, luteolin.

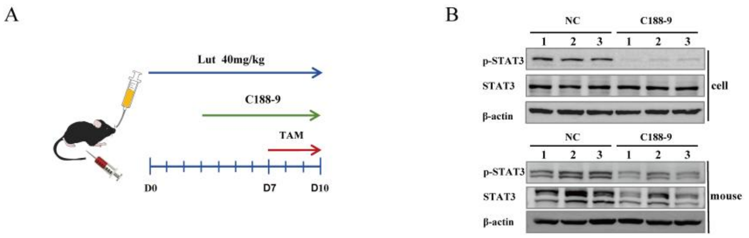

Figure S6. Validation of C188-9 efficiency. (A) Schematic image showing C188-9 and Lut for TAM-treated mice. (B) Western blotting for p-STAT3 and STAT3 in C188-9-treated cells and mice. NC, negative control.

Table S1. Detection conditions

Mass spectrum condition		Chromatographic condition	
Ion Source	Electrospray Ionization Source	Chromatographic column	XB-C18，50×2.1 mm，1.8 mm, Welch
Scanning mode	Positive and negative ion-switching scanning	Flow rate	0.30 mL/min
Detection method	Full mass/dd-MS2	Aqueous phase	0.1% formic acid aqueous solution
Resolution	70000 (full mass); 17500 (dd-MS2)	Organic phase	Methanol
Scan range	150.0–2000.0 m/z	Needle washing solution	Methanol
Spray Voltage	3.8 kV (Positive)	Column temperature box temperature	35 ºC
Capillary Temperature	300 ºC	Automatic injector temperature	10.0 ºC
Collision gas	High purity argon (purity ≥99.999%)	Automatic injector injection volume	5.00 μL
Sheath gas	Nitrogen (purity ≥99.999%), 40 Arb		
Aux gas heater temp	Nitrogen (purity ≥99.999%), 350 ºC		
Data collection time	30.0 min		

Table S2. Patient and histologic characteristics

Patient ID	Diagnosis Age (y)	Sex	Histologic characteristics (before treatment)	Histologic characteristics (after treatment)
P1	66	Male	(antrum) severe intestinal metaplasia with mild atypical hyperplasia;(angle)severe intestinal metaplasia with mild atypical hyperplasia	(antrum)moderate intestinal metaplasia with mild atypical hyperplasia; (angle)moderate intestinal metaplasia with mild atypical hyperplasia
P2	67	Male	(antrum) chronic inflammation of mucosal tissue with moderate intestinal metaplasia	(antrum)mild chronic inflammation, mild intestinal metaplasia
P3	62	Female	(antrum) moderate atrophy, mild intestinal metaplasia	(antrum)mild chronic atrophic inflammation, mild intestinal metaplasia
P4	60	Female	(posterior wall of antrum)mild chronic inflammation of superficial mucosa with intestinal metaplasia;(minor curvature of antrum)severe atrophic gastritis with severe intestinal metaplasia, focal mild dysplasia;(minor curvature of corpus)mild to moderate atrophic gastritis with mild to moderate intestinal metaplasia	(posterior wall of antrum)mild chronic atrophic gastritis; (minor curvature of antrum)mild chronic atrophic gastritis, moderate intestinal metaplasia, focal lymphocyte aggregation; (minor curvature of corpus)mild chronic atrophic gastritis, mild intestinal metaplasia
P5	39	Female	(pylorus, antrum)moderate to severe intestinal metaplasia	(minor curvature of antrum)mild chronic inflammation
P6	66	Female	(anterior antrum and cardiac)mild chronic gastritis, severe intestinal metaplasia of glands, severe atrophy, mucosal muscle hyperplasia	(antrum)moderate chronic inflammation of pyloric mucosa, moderate intestinal metaplasia
P7	42	Male	(antrum) moderate chronic atrophic gastritis with moderate intestinal metaplasia	(minor curvature of antrum) : mild chronic non-atrophic gastritis
P8	33	Female	(corpus)mild to moderate chronic inflammation and mild intestinal metaplasia	(angle) mild chronic superficial gastritis, moderate glandular atrophy; (antrum) mild chronic gastritis with mild intestinal metaplasia
P9	41	Male	(angle) moderate chronic atrophic inflammation, intestinal metaplasia; (minor curvature of antrum)mild chronic atrophic inflammation, intestinal metaplasia; (anterior wall of antrum) mild chronic atrophic inflammation, intestinal metaplasia	(angle) severe atrophic gastritis with severe intestinal metaplasia, mild dysplasia, and partial blockage of several small vascular lumens in deep mucosa; (minor curvature of antrum)moderate atrophic gastritis with severe intestinal metaplasia;(anterior wall of antrum) severe atrophic gastritis with severe intestinal metaplasia

P10	64	Female	(antrum) mild chronic inflammation of the gastric mucosa with severe intestinal metaplasia	(minor curvature of antrum)mild chronic inflammation of superficial mucosa with intestinal metaplasia;(posterior wall of antrum)mild chronic inflammation of superficial mucosa;(angle) severe atrophic gastritis with severe intestinal metaplasia, focal mild dysplasia, and individual glandular duct dilatation
P11	69	Male	(corpus)mild chronic inflammation with moderate intestinal metaplasia	(minor curvature of bottom corpus)mild chronic inflammation of mucosa, severe intestinal metaplasia; (minor curvature of middle corpus)mild chronic inflammation of mucosa and severe intestinal metaplasia
P12	66	Female	(greater curvature of antrum)mild chronic superficial gastritis with low-grade dysplasia of some glandular epithelium	(greater curvature of stomach)mild chronic superficial gastritis, some glands showed low grade dysplasia

Table S3. Cellmarkers

Cell group	Marker gene	Epithelial subgroup	Marker gene
B cell	CD79A, MS4A1	MUC5AC+ TFF1+	MUC5AC, TFF1
Endothelial cell	VWF	MUC6+	MUC6
Epithelial cell	EPCAM	Parietal cell	GIF, ATP4A
Fibroblast	DCN	Chief cell	LIPF
Mast cell	TPSAB1	Proliferative cell	PCNA, MKI67
Myeloid cell	CD14, CD68	Enteroendocrine cell	CHGA, CHGB
Plasma cell	CD79A, IGHG		
T&NK	CD3D, IL7R		
